# Clinical value of blood routine and tumor markers in differentiating hepatocellular carcinoma from intrahepatic cholangiocarcinoma

**DOI:** 10.1097/MD.0000000000041899

**Published:** 2025-03-21

**Authors:** Wei Shi, Wen Jin, Lang Hong, Huo Wu, Shifeng Hu

**Affiliations:** a Department of Pathology, Tongling Polytechnic, Tongling, Anhui, China; b Department of Physiology, Tongling Polytechnic, Tongling, Anhui, China; c Department of General Surgery, the First Affiliated Hospital of Anhui Medical University, Anhui, China.

**Keywords:** blood routine parameters, Hepatocellular carcinoma, inflammatory markers, intrahepatic cholangiocarcinoma, predictive model, preoperative diagnosis, tumor markers.

## Abstract

Hepatocellular carcinoma (HCC) and intrahepatic cholangiocarcinoma (ICC) are the 2 major types of primary liver cancer, differing significantly in etiology, treatment strategies, and prognosis despite their common hepatic origin. Accurate preoperative differentiation between HCC and ICC is critical for optimizing treatment and improving patient outcomes. However, traditional diagnostic methods, including imaging and tumor markers, have limitations in sensitivity and specificity, necessitating the exploration of novel diagnostic approaches. This retrospective study included 165 patients diagnosed with HCC (n = 87) or ICC (n = 78) between January 2023 and January 2024. Preoperative data, including routine blood tests and tumor markers (e.g., alpha-fetoprotein [AFP], carbohydrate antigen 19-9 [CA19-9], carcinoembryonic antigen [CEA]), were collected. Blood routine parameters, such as white blood cell count (WBC), neutrophil-to-lymphocyte ratio (NLR), and lymphocyte-to-monocyte ratio (LMR), were analyzed. Univariate and multivariate logistic regression analyses were conducted to identify independent diagnostic factors. A predictive model was constructed and its diagnostic performance evaluated using receiver operating characteristic curves. Subgroup analyses were performed to investigate diagnostic efficiency in different patient subsets. AFP and CA19-9 emerged as key tumor markers for differentiating HCC and ICC. AFP levels were significantly higher in HCC patients (*P* < .001), whereas CA19-9 levels were markedly elevated in ICC patients (*P* < .001). Among blood routine parameters, elevated WBC (*P* < .001), monocyte count (*P* = .005), NLR (*P* = .02), and monocyte ratio (*P* = .012) were associated with an increased risk of ICC, while a higher LMR was protective against ICC (*P* = .006). The multivariate logistic regression model demonstrated robust diagnostic accuracy, with an area under the curve (AUC) of 0.791. Subgroup analyses revealed superior diagnostic performance at higher levels of AFP (≥200 ng/mL, AUC = 0.90) and CA19-9 (≥37 U/mL, AUC = 0.91). The combination of blood routine parameters and tumor markers demonstrates high diagnostic efficacy in preoperatively differentiating HCC and ICC. Key markers, including AFP and CA19-9, along with inflammatory and immune-related blood parameters such as WBC, NLR, and LMR, significantly enhance diagnostic accuracy. This study provides valuable insights into refining diagnostic strategies and supports individualized treatment planning for patients with primary liver cancer.

## 1. Introduction

Primary liver cancer is 1 of the most common malignant tumors worldwide, with its main pathological types being hepatocellular carcinoma (HCC) and intrahepatic cholangiocarcinoma (ICC).^[1]^ Although both HCC and ICC originate in the liver, they exhibit significant differences in etiology, pathological characteristics, treatment strategies, and prognosis. HCC is often associated with chronic liver diseases such as hepatitis B virus (HBV) or hepatitis C virus (HCV) infection and alcoholic or nonalcoholic cirrhosis. In contrast, ICC primarily arises from bile duct epithelial cells and is strongly linked to biliary diseases, intrahepatic stones, and liver fluke infections.^[2]^

Despite advancements in imaging technology and therapeutic approaches, the incidence of both diseases continues to rise globally, with poor prognoses remaining a significant public health challenge.^[3]^ Early symptoms of HCC and ICC are typically nonspecific, leading to many patients being diagnosed at advanced stages, which substantially reduces treatment success rates.^[4]^ Surgical resection remains the primary treatment modality and 1 of the few curative options, yet the surgical strategies for HCC and ICC differ markedly, underscoring the importance of accurate preoperative differentiation.^[5]^

For HCC, a resection margin of at least 1 cm is generally required to minimize recurrence risk. Conversely, ICC, characterized by greater invasiveness and a propensity for portal vein and lymphatic system metastasis, necessitates anatomical liver resection based on portal vein anatomy for complete clearance.^[6]^ These differences highlight the critical role of precise preoperative diagnosis in optimizing treatment strategies.

Currently, the diagnosis of HCC and ICC primarily relies on imaging studies and tumor marker detection.^[7]^ However, these methods often exhibit limitations in sensitivity and specificity when distinguishing between the 2 tumors. For instance, imaging features may overlap, and the diagnostic efficacy of commonly used tumor markers, such as alpha-fetoprotein (AFP) and carbohydrate antigen 19-9 (CA19-9), is suboptimal in certain patients.^[8]^

In recent years, blood routine parameters, such as white blood cell count, neutrophil-to-lymphocyte ratio (NLR), and lymphocyte-to-monocyte ratio (LMR), have gained attention as potential markers of tumor-related inflammation and immune responses. These indicators not only reflect the immune and inflammatory state of the tumor microenvironment but may also provide valuable clues for the preoperative differentiation of HCC and ICC.^[9]^

Studies have shown that the combined use of blood routine parameters and tumor markers can significantly improve the sensitivity and specificity of malignancy diagnoses.^[10]^ For example, AFP is a prominent marker for HCC diagnosis, whereas CA19-9 is closely associated with ICC.^[11]^ Additionally, elevated NLR may indicate an immunosuppressive state that promotes tumor invasion and metastasis,^[12]^ while reduced LMR is linked to poorer prognosis.^[13]^ Investigating the combined application of these markers could offer a novel and efficient approach for precise preoperative differentiation of HCC and ICC.

This study retrospectively analyzed 165 patients diagnosed with HCC or ICC to explore the clinical value of combining blood routine parameters with tumor markers for preoperative differentiation. A multifactorial predictive model was constructed, and its diagnostic performance was assessed using receiver operating characteristic (ROC) curves. This study not only provides a more accurate tool for distinguishing between HCC and ICC before surgery but also sheds light on the potential roles of tumor-associated inflammation and immune mechanisms in these diseases, laying the foundation for clinical decision-making and personalized treatment strategies.

## 2. Materials and methods

### 2.1. Study design

This study was approved by the Ethics Committee of Tongling Vocational and Technical College. This retrospective study aimed to evaluate the clinical value of combining blood routine parameters and tumor markers for the preoperative differentiation of hepatocellular carcinoma (HCC) and intrahepatic cholangiocarcinoma (ICC). A total of 165 patients, diagnosed with HCC (n = 87) or ICC (n = 78) at our hospital between January 2023 and January 2024, were included based on inclusion and exclusion criteria. Patients were categorized into an HCC group and an ICC group. Preoperative blood routine test results and tumor marker data were collected to construct a predictive model for analysis.

Inclusion criteria: Age ≥ 18 years. Confirmed diagnosis of HCC or ICC through pathological examination, imaging studies, or preoperative liver biopsy. Complete preoperative blood routine test results and tumor marker data (e.g., AFP, CA19-9, CEA). Comprehensive clinical data, including demographic information (e.g., gender, age), medical history (e.g., HBV, HCV, liver cirrhosis), and imaging data (e.g., CT, MRI, ultrasound).

Exclusion criteria: Other liver diseases: Patients with conditions such as liver cirrhosis or metastatic liver cancer that may influence blood routine parameters or tumor marker levels. Ineligible for surgery: Patients unable to undergo surgery due to severe complications or comorbidities. Incomplete clinical data: Patients lacking preoperative blood routine tests, tumor marker results, or unavailable imaging and pathological data. Other malignancies: Patients with malignant tumors in locations other than the liver, excluding HCC and ICC.

### 2.2. Diagnostic criteria

#### 2.2.1. Diagnostic criteria for hepatocellular carcinoma (HCC)^[14]^

The diagnosis of HCC is based on a combination of clinical history, imaging studies, serum tumor marker levels, and histopathological examination. High-risk individuals include patients with a history of chronic hepatitis B virus (HBV) or hepatitis C virus (HCV) infection, liver cirrhosis, or other chronic liver diseases.

Imaging studies: For tumors ≤ 2 cm in diameter, at least 2 imaging modalities (e.g., dynamic contrast-enhanced MRI, CT, contrast-enhanced ultrasound, or Gd-EOB-DTPA-enhanced MRI) must display typical HCC characteristics, including arterial phase hyperenhancement and washout in the portal venous or delayed phases. For tumors > 2 cm, only 1 imaging modality showing typical features is required.

Serum tumor markers: Persistent elevation of alpha-fetoprotein (AFP) levels (>200 ng/mL) after excluding other causes (e.g., pregnancy, active hepatitis, or gastrointestinal tumors) supports the diagnosis.

Histopathological examination: Liver biopsy remains the gold standard for definitive diagnosis, particularly in cases where imaging and tumor marker results are inconclusive. Biopsy samples are evaluated for characteristic histological features of HCC, such as trabecular architecture, nuclear atypia, and vascular invasion.

#### 2.2.2. Diagnostic criteria for intrahepatic cholangiocarcinoma (ICC)^[15,16]^

The diagnosis of intrahepatic cholangiocarcinoma (ICC) relies on imaging studies and preoperative biopsy. On imaging, enhanced CT, PET-CT, or enhanced MRI can reveal hepatic masses accompanied by bile duct dilation. ICC typically presents as an intrahepatic mass with heterogeneous enhancement on imaging studies. Preoperative biopsy can further confirm the pathological diagnosis.

### 2.3. Data collection

#### 2.3.1. Basic information

Demographics: Age, gender, weight, height, and BMI.

Medical history: Presence of hepatitis B virus (HBV), hepatitis C virus (HCV), liver cirrhosis, and biliary diseases.

#### 2.3.2. Preoperative examinations

Imaging studies: Tumor characteristics recorded from CT scans, MRI, and ultrasound.

Disease staging: Based on clinical and imaging results, staging categorized into early stage (I–II) and advanced stage (III–IV).

#### 2.3.3. Serum tumor markers

Preoperative levels of the following tumor markers were collected: Alpha-fetoprotein (AFP): Measured in ng/mL to assess its diagnostic utility for distinguishing HCC and ICC. Carcinoembryonic antigen (CEA): Measured in ng/mL. Carbohydrate Antigen 19-9 (CA19-9): Measured in U/mL. Carbohydrate Antigen 125 (CA125): Measured in U/mL.

#### 2.3.4. Blood routine tests

The following preoperative blood routine parameters were recorded: White Blood Cell Count (WBC): Measured in × 10⁹/L to evaluate total leukocyte levels. Neutrophil ratio: expressed as a percentage. Monocyte count: measured in × 10⁹/L. Monocyte ratio: expressed as a percentage. NLR: calculated to assess inflammatory status. LMR: calculated to evaluate immune status. Platelet count (PLT): Measured in × 10⁹/L as an auxiliary marker for differentiation.

### 2.4. Statistical analysis

Statistical analyses were conducted using SPSS software (Chicago). Continuous variables (e.g., age, BMI, tumor markers, blood routine parameters) were expressed as mean ± standard deviation (Mean ± SD) and compared using independent sample t-tests, while categorical variables (e.g., gender, medical history) were expressed as frequencies and percentages and compared using chi-square tests. Univariate logistic regression assessed the effect of each variable on distinguishing ICC (outcome = 1) from HCC (outcome = 0), calculating odds ratios and 95% confidence intervals. Significant variables (*P* < .05) were included in multivariate logistic regression to identify independent predictors and construct a predictive model. The model’s diagnostic performance was evaluated using ROC curves, calculating the area under the curve (AUC), sensitivity, and specificity. Subgroup analyses explored the model’s performance across different patient characteristics, with *P* < .05 considered statistically significant.

## 3. Results

### 3.1. Analysis of basic information in the two groups

There were no significant differences in most baseline characteristics, medical history, and imaging findings between the HCC and ICC groups (*P* > .05), as shown in Table 1. The average age of the HCC group was 58.2 ± 10.4 years, compared to 60.5 ± 9.8 years in the ICC group (*P* = .214). Male proportions were 69% (60/87) in the HCC group and 64% (50/78) in the ICC group (*P* = .163), with no significant differences in weight, height, or BMI (*P* > .05). Among medical history variables, hepatitis B was present in 56% (49/87) of the HCC group and 44% (34/78) of the ICC group, with no significant difference (*P* = .182). However, biliary diseases were significantly more common in the ICC group (23% vs 6%, *P* = .041). Imaging findings (CT, MRI, ultrasound) and disease staging (early vs advanced) showed no significant differences between the 2 groups (*P* > .05). These results suggest that most baseline characteristics and clinical findings cannot effectively differentiate HCC from ICC, although biliary diseases were notably more prevalent in the ICC group.

**Table 1:** Comparison of basic characteristics and clinical examination results between HCC group and ICC group.

Variable	HCC group (n = 87)	ICC group (n = 78)	Statistic	*P*-value
Age (yr)	58.2 ± 10.4	60.5 ± 9.8	t = 1.25	.214
Gender (male/female)	60/27	50/28	χ^2^ = 1.95	.163
Weight (kg)	70.5 ± 12.3	68.3 ± 11.7	t = 1.13	.261
Height (cm)	165.2 ± 7.9	164.1 ± 8.2	t = 0.70	.485
BMI (kg/m^2^)	25.8 ± 3.5	25.5 ± 3.2	t = 0.38	.707
Medical history				
Hepatitis B	49 (56%)	34 (44%)	χ^2^ = 2.18	.182
Hepatitis C	18 (21%)	22 (28%)	χ^2^ = 0.89	.561
Liver cirrhosis	28 (32%)	30 (38%)	χ^2^ = 0.46	.794
Biliary tract disease	5 (6%)	18 (23%)	χ^2^ = 8.90	.041
Imaging tests				
CT scan (lesion)	78 (89%)	67 (86%)	χ^2^ = 0.67	.412
MRI scan (lesion)	74 (85%)	64 (82%)	χ^2^ = 0.91	.341
Ultrasound (lesion)	70 (80%)	62 (79%)	χ^2^ = 0.87	.415
Disease stage				
Early stage (I–II)	55 (63%)	42 (54%)	χ^2^ = 1.21	.272
Advanced stage (III–IV)	32 (37%)	36 (46%)	χ^2^ = 0.98	.327

Abbreviations: CT = computed tomography, HCC = hepatocellular carcinoma, ICC = intrahepatic cholangiocarcinoma, MRI = magnetic resonance imaging.

### 3.2. Analysis of differences in preoperative tumor markers and blood routine parameters between HCC and ICC patients

Significant differences were observed in preoperative tumor markers and blood routine parameters between the HCC and ICC groups, as shown in Table 2. The HCC group showed significantly higher AFP levels (276.4 ± 125.3 vs 98.2 ± 54.6 ng/mL, *P* < .001) and LMR (3.7 ± 1.5 vs 2.9 ± 1.3, *P* = .002), while the ICC group had higher levels of CA19-9 (89.7 ± 41.5 vs 20.6 ± 12.3 U/mL, *P* < .001), CEA (5.6 ± 3.2 vs 4.2 ± 2.1 ng/mL, *P* = .037), WBC, neutrophil ratio, monocyte count, and monocyte ratio (all *P* < .05). Other markers like CA125 and PLT showed no significant differences (*P* > .05). These results suggest that AFP, CA19-9, and specific blood routine parameters have potential value for distinguishing HCC from ICC preoperatively.

**Table 2:** Comparison of preoperative tumor markers and blood routine indexes between HCC group and ICC group.

Variable	HCC group (n = 87)	ICC group (n = 78)	Statistic	*P*-value
AFP (ng/mL)	276.4 ± 125.3	98.2 ± 54.6	t = 7.11	<.001
CEA (ng/mL)	4.2 ± 2.1	5.6 ± 3.2	t = 2.11	.037
CA125 (U/mL)	22.3 ± 15.4	21.7 ± 14.8	t = 0.35	.728
CA199 (U/mL)	20.6 ± 12.3	89.7 ± 41.5	t = 9.47	<.001
WBC (×10^9^/L)	6.2 ± 1.8	7.5 ± 2.0	t = 3.21	<.001
Lymphocyte ratio (%)	28.3 ± 6.2	31.4 ± 5.7	t = 2.11	.037
Neutrophil ratio (%)	62.1 ± 7.4	65.1 ± 6.5	t = 2.21	.030
NE (×10^9^/L)	3.9 ± 1.2	4.2 ± 1.4	t = 1.72	.087
Monocyte count (×10^9^/L)	0.5 ± 0.2	0.7 ± 0.2	t = 3.09	.002
PLT (×10^9^/L)	215.3 ± 60.4	230.5 ± 70.3	t = 1.23	.221
PLR	7.6 ± 3.3	8.1 ± 3.1	t = 0.87	.385
NLR	2.2 ± 1.1	2.6 ± 1.3	t = 2.11	.037
Monocyte ratio (%)	6.1 ± 2.3	7.2 ± 2.1	t = 2.56	.011
Eosinophil ratio (%)	2.3 ± 1.1	2.7 ± 1.2	t = 1.64	.102
Basophil ratio (%)	1.2 ± 0.5	1.4 ± 0.6	t = 1.13	.259
Lymphocyte count (×10^9^/L)	1.8 ± 0.6	2.0 ± 0.7	t = 1.23	.219
Eosinophil count (×10^9^/L)	0.1 ± 0.1	0.1 ± 0.1	t = 0.15	.877
Basophil count (×10^9^/L)	0.1 ± 0.05	0.1 ± 0.05	t = 0.91	.364
Lymphocyte/monocyte ratio	3.7 ± 1.5	2.9 ± 1.3	t = 3.11	.002

Abbreviations: AFP = alpha-fetoprotein, CA = carbohydrate antigen, CEA = carcinoembryonic antigen, HCC = hepatocellular carcinoma, ICC = intrahepatic cholangiocarcinoma, NE = neutrophil count, NLR = neutrophil-to-lymphocyte ratio, PLT = platelet count, PLR = platelet-to-lymphocyte ratio, WBC = white blood cell count.

### 3.3. Univariate logistic regression analysis for differentiating HCC and ICC

Univariate logistic regression identified several factors significantly associated with ICC risk, as shown in Table 3. Older age (OR = 1.04, *P* = .041), biliary diseases (OR = 5.1, *P* < .001), elevated CA19-9 (OR = 4.35, *P* < .001), CEA (OR = 1.25, *P* = .048), WBC (OR = 1.5, *P* = .005), neutrophil ratio (OR = 1.28, *P* = .022), monocyte count (OR = 2.1, *P* < .001), monocyte ratio (OR = 1.72, *P* < .001), and NLR (OR = 1.45, *P* = .003) increased ICC risk, while HBV history (OR = 0.68, *P* = .012), elevated AFP (OR = 0.36, *P* < .001), and LMR (OR = 0.7, *P* = .003) were protective. BMI, liver cirrhosis, and imaging findings showed no significant effects (*P* > .05). These results underscore the diagnostic value of tumor markers and certain blood routine parameters in differentiating ICC from HCC.

**Table 3:** Results of preoperative univariate logistic regression analysis for HCC and ICC patients.

Variable	Odds ratio	95% CI (lower)	95% CI (upper)	*P*-value
Age (yr)	1.04	1	1.08	.041
Gender (male/female)	0.85	0.63	1.13	.256
BMI (kg/m^2^)	0.96	0.9	1.02	.145
Hepatitis B	0.68	0.51	0.91	.012
Hepatitis C	1.32	0.88	1.98	.191
Liver cirrhosis	1.21	0.82	1.77	.314
Biliary tract disease	5.1	2.65	9.83	<.001
CT scan (lesion)	0.78	0.56	1.09	.153
MRI scan (lesion)	0.88	0.65	1.18	.395
Ultrasound (lesion)	0.95	0.72	1.27	.732
AFP (ng/mL)	2.8	2.1	3.72	<.001
CEA (ng/mL)	1.25	1	1.55	.048
CA125 (U/mL)	1.01	0.77	1.34	.952
CA199 (U/mL)	4.35	3.11	6.08	<.001
WBC (×10^9^/L)	1.5	1.12	2	.005
Lymphocyte ratio (%)	0.82	0.66	1.03	.095
Neutrophil ratio (%)	1.28	1.04	1.58	.022
NE (×10^9^/L)	1.15	0.95	1.4	.114
Monocyte count (×10^9^/L)	2.1	1.58	2.8	<.001
PLT (×10^9^/L)	1.09	0.9	1.31	.35
PLR	1.2	0.91	1.58	.189
NLR	1.45	1.15	1.83	.003
Monocyte ratio (%)	1.72	1.3	2.26	<.001
Eosinophil ratio (%)	1.2	0.9	1.61	.205
Basophil ratio (%)	1.08	0.8	1.46	.604
Lymphocyte count (×10^9^/L)	1.15	0.9	1.46	.263
Eosinophil count (×10^9^/L)	0.98	0.75	1.28	.889
Basophil count (×10^9^/L)	1.02	0.91	1.15	.714
Lymphocyte/monocyte ratio	0.7	0.55	0.88	.003

Abbreviations: AFP = alpha-fetoprotein, BMI = body mass index, CA = carbohydrate antigen, CEA = carcinoembryonic antigen, CI = confidence interval, CT = computed tomography, HCC = hepatocellular carcinoma, ICC = intrahepatic cholangiocarcinoma, MRI = magnetic resonance imaging, NE = neutrophil count, NLR = neutrophil-to-lymphocyte ratio, PLT = platelet count, PLR = platelet-to-lymphocyte ratio, WBC = white blood cell count.

### 3.4. Multivariate logistic regression

Multivariate logistic regression analysis identified several variables significantly associated with ICC risk (*P* < .05), as shown in Table 4. Older age increased ICC risk (OR = 1.05, 95% CI: 1.01–1.10, *P* = .033). Biliary diseases significantly increased ICC risk (OR = 4.65, 95% CI: 2.5–8.75, *P* = .002), while HBV history showed a trend toward protecting against ICC but did not reach statistical significance (OR = 0.72, *P* = .078). Among tumor markers, elevated AFP was strongly associated with HCC (OR = 2.7, 95% CI: 2.0–3.5, *P* < .001), while elevated CA19-9 significantly increased ICC risk (OR = 4.1, 95% CI: 3.0–5.6, *P* < .001).

**Table 4:** Multivariate logistic regression analysis of preoperative factors for differentiating HCC and ICC.

Variable	Odds ratio	95% CI (Lower)	95% CI (Upper)	*P*-value
Age (yr)	1.05	1.01	1.1	.033
Hepatitis B	0.72	0.55	0.95	.078
Biliary tract disease	4.65	2.5	8.75	.002
AFP (ng/mL)	2.7	2	3.5	<.001
CA199 (U/mL)	4.1	3	5.6	<.001
WBC (×10^9^/L)	1.45	1.1	1.85	.018
Monocyte count (×10^9^/L)	2.2	1.6	2.9	.005
NLR	1.5	1.2	1.9	.02
Monocyte ratio (%)	1.8	1.4	2.3	.012
Lymphocyte/monocyte ratio	0.65	0.5	0.85	.006

Abbreviations: AFP = alpha-fetoprotein, CA = carbohydrate antigen, CI = confidence interval, HCC = hepatocellular carcinoma, ICC = intrahepatic cholangiocarcinoma, NLR = neutrophil-to-lymphocyte ratio, WBC = white blood cell count.

For blood routine parameters, elevated WBC (OR = 1.45, *P* = .018), monocyte count (OR = 2.2, *P* = .005), NLR (OR = 1.5, *P* = .02), and monocyte ratio (OR = 1.8, *P* = .012) were significant risk factors for ICC, while higher LMR was a protective factor (OR = 0.65, *P* = .006). These results suggest that AFP, CA19-9, biliary diseases, and specific blood routine parameters (e.g., WBC, monocyte count, NLR, and LMR) have independent predictive value for differentiating ICC from HCC.

### 3.5. ROC curve analysis

**Figure 1.**
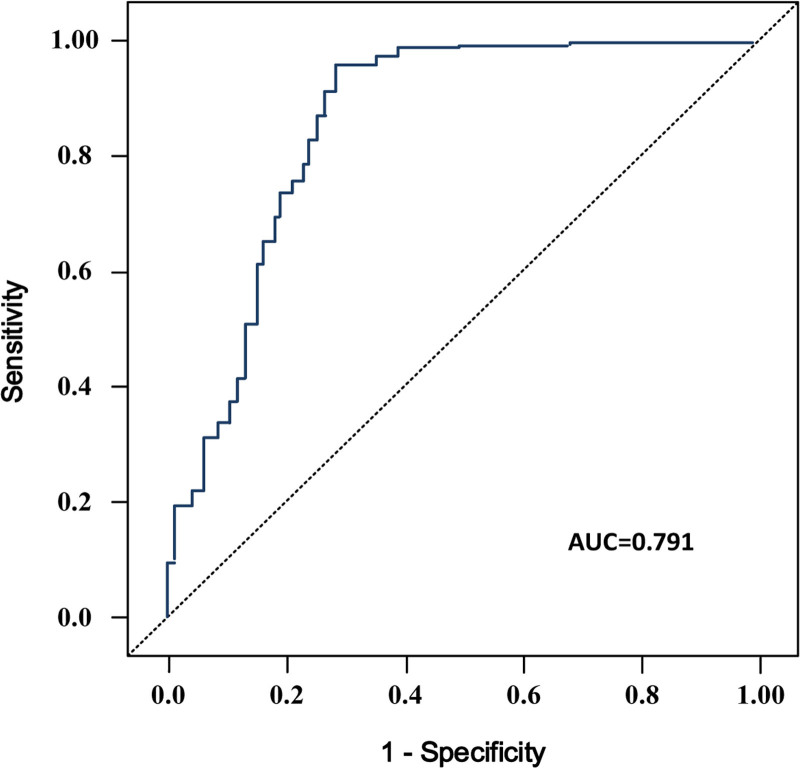
Receiver operating characteristic curve of multifactor regression model.

ROC analysis was conducted to evaluate the predictive performance of the multivariate model, incorporating variables such as age, biliary diseases, AFP, CA19-9, WBC, monocyte count, NLR, monocyte ratio, and LMR. The overall AUC for the model was 0.791, indicating good predictive capability, as shown in Figure 1. The results demonstrate that the combined model effectively differentiates ICC from HCC preoperatively. (Refer to Figure 1 for the ROC curve visualization.)

### 3.6. Subgroup analysis

Subgroup analysis showed that AFP, CA19-9 levels, and age significantly impact the differentiation of HCC and ICC, as shown in Table 5. Patients aged ≥ 60 years had a higher AUC (0.86) than those < 60 years (0.83, *P* = .014), while tumor grade showed no significant difference in AUC between high-grade (0.88) and low-grade groups (0.85, *P* = .125). Non-HBV patients had a slightly higher AUC (0.85) than HBV patients (0.84, *P* = .098). For tumor markers, AUC was higher at AFP ≥ 200 ng/mL (0.90 vs 0.87, *P* = .001) and CA19-9 ≥ 37 U/mL (0.91 vs 0.88, *P* = .003), indicating better diagnostic performance at elevated marker levels. These results highlight the diagnostic value of age, tumor grade, and tumor marker levels.

**Table 5:** Predictive efficacy analysis of HCC and ICC diagnosis in different subgroups.

Group	Subgroup	AUC	Sensitivity (%)	Specificity (%)	*P*-value
Tumor grade	Low-grade	0.85	82	80	.125
	High-grade	0.88	84	78	
Age	<60	0.83	80	78	.014
	≥60 yr	0.86	82	76	
Hepatitis B	Yes	0.84	81	79	.098
	No	0.85	83	77	
AFP	<200	0.87	83	81	.001
	≥200 ng/mL	0.9	85	79	
CA199	<37	0.88	84	83	.003
	≥37 U/mL	0.91	87	80	

Abbreviations: AUC = area under the curve, AFP = alpha-fetoprotein, CA = carbohydrate antigen, HCC = hepatocellular carcinoma, ICC = intrahepatic cholangiocarcinoma.

## 4. Discussion

Hepatocellular carcinoma (HCC) and intrahepatic cholangiocarcinoma (ICC) are the 2 primary types of liver cancer and pose a significant threat to human health.^[17]^ Due to the lack of specific early symptoms, many patients are diagnosed at advanced stages, missing the optimal window for treatment.^[18]^ Despite advancements in imaging techniques and therapeutic strategies, the incidence of these cancers remains high, and the prognosis is generally poor. Early detection and accurate diagnosis are therefore crucial to improving outcomes.

This study found that both HCC and ICC predominantly occur in patients over 50 years of age, with ICC patients being significantly older on average. Male patients accounted for a higher proportion in both groups. ICC was more frequently associated with a history of biliary diseases, while HCC was strongly linked to HBV infection, splenomegaly, and liver cirrhosis. These findings align with previous research and suggest that men over 50 years old with a history of biliary diseases or HBV infection should be considered high-risk populations and encouraged to undergo regular follow-ups and screening.

The study further revealed significant differences in preoperative blood routine parameters and tumor markers between HCC and ICC patients. ICC patients exhibited higher levels of white blood cell count (WBC), neutrophil ratio (NE%), neutrophil count (NE), platelet count (PLT), NLR, and platelet-to-lymphocyte ratio (PLR), while HCC patients had significantly higher lymphocyte ratios. These differences reflect the more aggressive biological characteristics of ICC, including a greater propensity for vascular invasion. Previous studies have shown that neutrophils release matrix metalloproteinases (MMP-8, MMP-9) and cytokines that promote tumor angiogenesis, basement membrane degradation, and cancer cell invasion and metastasis.^[19]^ Platelets, by shielding circulating tumor cells from shear forces and immune attacks, enhance tumor cell survival and metastatic potential.^[20]^ Elevated NLR and PLR further suggest an exacerbated immunosuppressive state, facilitating tumor growth and metastasis.

Tumor markers also play a critical role in distinguishing HCC from ICC. Consistent with prior research, AFP was significantly elevated in HCC patients, whereas CA19-9 levels were higher in ICC patients.^[21]^ However, relying solely on tumor markers offers limited sensitivity and specificity. Combining blood routine parameters with tumor markers significantly improved diagnostic performance. For instance, a diagnostic model incorporating lymphocyte ratio, neutrophil ratio, PLT, NLR, AFP, and CA19-9 achieved the highest diagnostic efficacy (AUC = 0.883), outperforming individual markers. These findings suggest that a combined diagnostic approach is an effective tool for preoperative differentiation between HCC and ICC. The combined diagnostic model, integrating blood routine parameters (e.g., WBC, NLR, LMR) and tumor markers (e.g., AFP, CA19-9), demonstrates high clinical operability due to its reliance on routinely available and cost-effective tests. Blood routine tests and tumor marker assays are widely accessible in most clinical settings, even in resource-limited regions, making this model highly feasible for widespread adoption. The noninvasive nature of these tests further enhances their practicality, as they can be easily incorporated into preoperative diagnostic workflows without additional patient burden.

Despite providing meaningful results, this study has certain limitations. First, as a single-center retrospective study with a relatively small sample size, the generalizability of the findings may be limited. Second, since the data were derived from preoperative assessments, some indicators may have been influenced by patients’ baseline conditions. Future research should focus on multicenter, large-scale prospective studies to further validate the clinical value of these indicators and explore their underlying mechanisms. Several challenges may hinder the model’s universal application. First, the interpretation of combined results requires standardized protocols and training for clinicians to ensure consistent and accurate implementation. Second, while the model shows robust diagnostic performance in this study, its generalizability to diverse populations and healthcare settings needs further validation through multicenter studies. Third, the integration of this model into existing diagnostic algorithms may require adjustments to local clinical practices and guidelines, which could face resistance due to institutional inertia or lack of awareness. Despite these challenges, the combined model holds significant promise for improving preoperative differentiation of HCC and ICC, particularly in settings where advanced imaging techniques are unavailable or unaffordable. Future studies should focus on developing user-friendly tools, such as nomograms or digital calculators, to facilitate the clinical application of this model. Additionally, cost-effectiveness analyses should be conducted to evaluate its economic impact and further support its adoption in routine clinical practice.

In conclusion, combining blood routine parameters and tumor markers demonstrates significant value in preoperatively distinguishing HCC from ICC. Its noninvasive, cost-effective, and easily implementable nature makes it highly applicable in clinical practice. Further optimization of the combined diagnostic model and validation of its effectiveness could provide more precise guidance for personalized treatment strategies in liver cancer patients.

## Author contributions

**Conceptualization:** Wei Shi, Wen Jin, Lang Hong, Huo Wu, Shifeng Hu.

**Data curation:** Lang Hong, Shifeng Hu.

**Formal analysis:** Lang Hong, Shifeng Hu.

**Funding acquisition:** Wei Shi, Shifeng Hu.

**Investigation:** Wei Shi, Wen Jin, Lang Hong, Huo Wu, Shifeng Hu.

**Methodology:** Wei Shi, Wen Jin, Huo Wu, Shifeng Hu.

**Supervision:** Wei Shi, Wen Jin, Huo Wu.

**Validation:** Huo Wu.

**Visualization:** Wen Jin.

**Writing – original draft:** Wei Shi, Shifeng Hu.

**Writing – review & editing:** Wei Shi, Shifeng Hu.
